# The hidden burden: self-reported irritability in adolescent girls signals higher psychiatric risk

**DOI:** 10.1186/s12889-025-23076-6

**Published:** 2025-05-19

**Authors:** Pablo Vidal-Ribas, Georgina Krebs, Jamilah Silver, Wan-Ling Tseng, Tamsin Ford, Argyris Stringaris

**Affiliations:** 1 Child and Adolescent Mental Health Research Group, Institut de Recerca Sant Joan de Déu, Esplugues de Llobregat, Barcelona, Spain; 2 Research Department of Clinical, Education and Health Psychology, University College London, London, UK; 3 Department of Psychology, Stony Brook University, Stony Brook, NY USA; 4 Yale Child Study Center, Yale School of Medicine, New Haven, CT USA; 5 Department of Psychiatry, University of Cambridge, Cambridge, UK; 6 Cambridge and Peterborough NHS Foundation Trust, Cambridge, UK; 7 Anxiety Self-Image and Mood (AIM) Laboratory, Division of Psychiatry and Psychology and Language Sciences, University College London, London, UK; 8 Department of Psychiatry, National and Kapodistrian University of Athens, Athens, Greece

**Keywords:** Irritability, Sex difference, Adolescent, Female, Informant

## Abstract

**Background:**

Most research on pediatric irritability focuses on children and/or relies on parent reports. We examined how self-reported irritability in adolescents influences the prevalence, sex distribution and correlates of irritability relative to children and parent reports.

**Methods:**

Using data from Mental Health of Children and Young People Survey 2017 in England we contrasted the prevalence of irritability, encompassing irritable mood and temper outbursts, in 2,740 adolescents aged 12–17 (50.3% females), based on parent- and self-report, with that of 4,141 children aged 5–11 (49.4% females) based on parent-report. We examined associations of irritability with mental health problems and impairment.

**Results:**

Parents reported similar prevalence of irritability in adolescent males (14–23%) and females (14–22%), but higher levels of irritability in males (20–25%) than females (15–19%) during childhood. In contrast, adolescent females self-reported more irritable mood (29%, 95%CI 26–31) than males (23%, 95%CI 20–25) and parents. Self-reported irritability in adolescent females was associated with greater emotional problems (irritable mood, b = 0.27, SE = 0.10, *p* = 0.011; temper outbursts: b = 0.25, SE = 0.11, *p* = 0.022) and impairment (irritable mood, b = 0.31, SE = 0.10, *p* = 0.001; temper outbursts: b = 0.31, SE = 0.08, *p* < 0.001) compared to males. Irritable mood in adolescent females was associated with a higher increase of psychiatric disorders (b = 0.35, SE = 0.15, *p* = 0.020) compared to males.

**Conclusions:**

Age, sex, and informant are sources of heterogeneity in irritability reporting, and must be considered in the assessment and understanding of irritability-related psychopathology. Longitudinal design studies with comprehensive assessments of irritability across a broad age range are warranted to elucidate its developmental trajectory and causal relationships with other psychopathological symptoms.

**Supplementary Information:**

The online version contains supplementary material available at 10.1186/s12889-025-23076-6.

## Background

Irritability is defined as proneness to anger that may reach a pathological extent [1]. It often accompanies both externalizing and internalizing mental health problems, and it is one of the most common symptoms for which families seek mental health treatment [2]. Whilst considerable progress has been made in understanding the developmental origins of irritability [3] and its mechanistic underpinnings [4], we still lack basic understanding of how factors such as age, sex, and informant influence the reported prevalence of, and impairment due to, irritability in youth. Limited knowledge on these important sources of heterogeneity hampers mechanistic research and prevents rational intervention. This is especially true during adolescence, a period in which irritability is largely understudied, many mental health problems typically onset, and the mental health gap widens between males and females. This paper seeks to narrow an important knowledge gap by examining potential sources of heterogeneity in reported irritability in adolescents from a large representative population-based sample.

Over the past 15 years, epidemiological research on pediatric irritability has provided valuable insights [1]. However, most epidemiological studies cover preschool or middle-childhood [5–9], with few studies including adolescents [7, 10–13]. This limits our understanding of irritability expression and correlates in adolescence. In addition, most studies including adolescents relied solely on parent reports of irritability [7, 11, 13, 14], or on combined reports merging parent and self-reports using the “OR rule”, thus preventing the study of informant effects [7, 10, 15]. Among the few studies relying on adolescent reports separately [12, 16, 17], none compared self-reports with parent-reports or with reports provided at younger ages (either on the same children or others), and only one examined sex differences in self-reported irritability [17]. Ignoring reports from young people themselves might hinder a deeper understanding of irritability, since recent evidence suggests that parents and their children interpret irritability items differently [18] and the correlates of irritability might differ by informant [19, 20]. Indeed, the Lancet Commission on Adolescent Health and Wellbeing recommended giving adolescents a stronger voice in identifying their own relevant health issues [21].

Collecting self-reports of irritability might also be relevant for the study of sex differences in the transition from childhood to adolescence, a topic that has received very little study. In adolescence, sex disparities in mental health widen, with females experiencing worse mental health worldwide [22]. Moreover, in this developmental period, the likelihood of externalizing behavior decreases while that of internalizing behavior increases, meaning that parents may be less likely to know the child’s emotional state [23]. This is particularly true for adolescent females, e.g., data from the Millennium Cohort Study show that parents were more likely to report emotional problems in males – in contrast to behavior problems—than were the males themselves, whereas the opposite was seen for females, and the informant discrepancy was larger in females than males [24]. The gap between adolescent females’ mental health problems and their parents’ awareness has increased in recent years, with several reports pointing to a worsening of such problems in adolescent females that goes unnoticed by their parents [25, 26].

Whereas some results suggest no substantial difference in the levels of irritability between male and female adolescents [10, 27], other studies do find differences in irritability trajectories and correlates. For instance, parent-reported data from the Avon Longitudinal Study of Parents and Children (ALSPAC) cohort showed higher levels of irritability in adolescent females than males, whereas the latter showed higher levels in childhood [14]. Additionally, studies have found a female preponderance for an increasing trajectory class in the transition from childhood to adolescence [13], as well as distinct longitudinal associations by sex with externalizing and internalizing psychopathology, with stronger associations with internalizing symptoms in females [13, 19, 27]. Since most of these studies were based on parent reports, there is a need for more studies using self-reported irritability in adolescences to better understand informant effects, sex effects, and the burden of irritability in adolescent females.

Here, we explore the impact of sex and informant on reported irritability and associated impairment in adolescents from a large population-based cohort. To achieve this, we use the Mental Health survey from the UK Office for National Statistics, which in 2017 incorporated a comprehensive assessment of irritability by both informants (i.e., parents and youth), including the characterization of irritable mood and temper outbursts. Irritable mood and temper outbursts are the two core components of severe chronic irritability, according to DSM-5 criteria of disruptive mood dysregulation disorder (DMDD). Irritable mood is characterized by persistently angry, grumpy, or grouchy mood; whereas temper outbursts are conceptualized as behavioral or verbal outbursts of intense anger limited in time. There is some evidence to suggest that irritable mood is more common in adolescent females [13, 14], and temper outbursts are more common in males, at least in referred samples [28, 29]. Regardless, given the potential informant discrepancies across sexes mentioned earlier, this question requires further study using a multi-informant approach.

Our study has two primary objectives. First, we examine the prevalence of irritable mood and temper outbursts (together referred to as irritability hereafter) in adolescents aged 12–17 and test whether it differs by sex and informant, in addition to contrasting adolescent with childhood irritability. We are particularly interested in exploring sex differences in adolescence because, as stated above, irritability and its impact in females has been understudied and underappreciated, possibly due to higher rates of males with irritability in clinical samples [29]. Second, we examine how irritability, characterized by irritable mood and temper outbursts, is associated with psychopathological symptoms, psychiatric disorders, and impairment in adolescence, and how these associations contrast to those seen in childhood.

## Methods

### Participants

This study employs data from the Mental Health of Children and Young People Survey 2017 from the Office of National Statistics (UK) [30, 31]. Children and young people were eligible to participate if they were aged 2 to 19, lived in England, and were registered with a general practitioner (GP). In October 2016, a stratified multistage random probability sample of 18,029 children was drawn from the NHS Patient Register using a two-stage process: first, 380 postcode sectors (primary sampling units, PSUs) were randomly selected for the main sample and 80 for a reserve sample; second, 42 children were randomly selected within each sector from both samples. Of the 18,029 issued addresses, 393 (2%) were deemed ineligible (e.g., participant had moved and was untraceable), leaving 17,636 eligible addresses. Among these, 4,956 (28%) refused participation, 2,194 (12%) could not be contacted, and 1,369 (8%) were categorized as ‘other unproductive’ (including 1% due to language barriers). Ultimately, 9,117 households participated, each with one or more informant (Figure S1).

Parents and children aged 11 and older were interviewed face-to-face by trained lay interviewers using computer-assisted interviews, with self-completed sections for more sensitive topics such as self-harm, drug use, and sexual identity. For children aged 2 to 10, only the parent or legal guardian was interviewed. Additionally, teachers completed an online or paper questionnaire for children aged 5 to 16 when parental consent was given. In total, 8,602 parent interviews were completed for children aged 2–19, and 3,545 interviews were completed with children aged 11–19.

Data for the current study were derived from the DMDD section of the Development and Well-Being Assessment (DAWBA) [32], which starts with two skip questions about the frequency of irritability. Consistent with the DSM-5 age criteria [33], these questions were directed to both parent and children about all participants aged 5–17. Specifically, parent-reported data on the frequency of irritability was collected from 6,881 children aged 5–17 (49.8% females) and both parent- and self-reported data were collected from 2904 children aged 11–17 (50.7% females). In line with a developmental framework, we defined two age periods: childhood, spanning ages 5–11 (n = 4,141, 49.4% females), and adolescence, spanning ages 12 to 17 years. (2,740 adolescents, 50.3% females).

### Ethical considerations

The survey received ethical approval from the West London & GTAC Research Ethics Committee in April 2016 (REC reference: 16/LO/0155), with a substantial amendment approved in October 2016. It was also approved by the Health Research Authority Confidentiality Advisory Group in May 2016 (CAG reference: 16/CAG/0016), with a subsequent amendment approved in September 2016. Informed consent was obtained from parents of children aged 2–16, with assent provided by children aged 11–16, while participants aged 17–19 gave their own informed consent.

### Measures

#### Psychiatric disorders

The DAWBA [32] is a multi-informant standardized diagnostic assessment combining structured and semi-structured elements. After interviews were completed, trained clinical raters reviewed the response to both elements from parents, children and teachers to assign DSM-5 diagnoses. The kappa statistic for chance-corrected agreement between two clinical raters, which were estimated in the first survey, was 0.86 for any disorder [standard error (SE) = 0.04], 0.57 for internalizing disorders (SE = 0.11), and 0.98 for externalizing disorders (SE = 0.02) [34]. Our study examined associations of irritability with the overall presence of psychiatric disorder; externalizing disorders (the combination of conduct, oppositional defiant and attention-deficit hyperactivity disorders); and internalizing disorders (the combination of depressive and anxiety-related disorders).

#### Irritability

The DMDD section of the DAWBA starts with two skip questions directed to all informants about the frequency of irritable mood (i.e., *“Most young people are sometimes in a really irritable or angry mood. On average over the last 6 months how often [has your child/have you] been in an angry or irritable mood?”*) and temper outbursts (i.e., *“Most young people sometimes have temper outbursts when they are angry- for example, shouting or slamming doors. On average over the last 12 months, how often [has your child/have you] had a temper outburst?”*). Each of these questions is answered with a five-point Likert scale as follows: (0) Never, (1) Occasionally, (2) Once or twice a week, (3) Three or more times a week, or (4) Every day. For the current study, we operationalized children presenting irritable mood and temper outbursts as those scoring 2 or higher on these questions (i.e., at least once or twice a week), which is the threshold used by the DAWBA to screen for irritability and to keep inquiring about other DMDD criteria. We used these binary variables to estimate prevalences and generate plots. In all other analyses, the five-point Likert scale was used, treating it as a continuous variable.

#### General psychopathology and associated impairment

The Strengths and Difficulties Questionnaire (SDQ) was used to assess general psychiatric symptoms. It includes 25 items rated on a three-point Likert scale, indicating how much each attribute applies to the target child (or to the respondent in the self-report version) [35]. The SDQ consists of five subscales—emotional symptoms, conduct problems, hyperactivity–inattention, peer problems, and prosocial behaviour—with five items each. All subscales except prosocial behaviour are summed to create a total difficulties score ranging from 0 to 40. The SDQ also includes an impact supplement that asks whether the child or youth has a problem and, if so, assesses overall distress, social impairment, burden, and chronicity. The instrument has strong psychometric properties [36], with Cronbach’s alpha in our sample at 0.87 for parent reports and 0.82 for self-reports. In the current study, we examined associations of irritability with emotional, conduct, and hyperactivity-inattention problem scales, as well as the overall impact score.

#### Psychosocial impairment indicators

We also examined associations between irritability and the presence of other impairment indicators collected in the survey; these included whether the child ever self-harmed or made a suicide attempt (by both parent and self-report); whether the child had ever been excluded from school; and whether the child sought help in the past year for emotional, behavioral, or concentration difficulties (these by parent report, or self-report for those aged 17).

### Statistical analysis

The survey data were weighted to take account of selection probabilities and non-response, so that the results were representative of the population aged 2 to 19. More details about the creation of weights and calibration are available in the NHS Digital report [30] and briefly described in the supplemental material.

Using the binary variables of irritability, we estimated the prevalence of irritable mood and temper outbursts by sex and informant in adolescence, and compared these to the prevalence in childhood, which was based solely on parent reports. We then used linear regression with the five-point Likert scale of irritability as a continuous outcome to examine the effects of informant (only for adolescents), sex, and developmental period on the frequency of irritability. For parent-report, we examined age by sex interactions with frequency of irritability (either irritable mood or temper outbursts) as the outcome, and for self-report in adolescence, we only examined sex effects since self-report data were only available for young people aged 11 and older.

To explore informant and sex effects on irritability correlates in adolescence, we first examined the association between frequency of irritability and general psychopathology, including emotional problems, hyperactivity problems, conduct problems, and overall associated impairment. To do so, we performed separate linear regression analyses for each informant (parent- and self-report) and each type of irritability (irritable mood and temper outbursts). The predictors included irritability, using the five-point Likert scale, sex, and their interactions while the outcomes were the corresponding SDQ scales reported by the same informant. Cross-informant predictions were also examined and are reported in the supplemental information.

Next, we examined the prevalence of any psychiatric disorder, any internalizing disorder, and any externalizing disorder based on the presence of irritability (separately for irritable mood and temper outbursts) as reported by each informant and sex. Given the disparity of prevalence of psychiatric disorders between males and females based on type of disorder, we based the description of the results on sex ratios (male:female). We then conducted, for each informant, and for irritable mood and temper outbursts separately, a series of logistic regressions with irritability, using the five-point Likert scale, sex, and their interaction as predictors, and each group of disorders as outcome.

Finally, we examined informant and sex effects in the association between irritability and other indicators of psychosocial impairment, which included parent- and self-reported self-harm, school exclusion, and use of mental health services. To do so, we performed, for each informant, and for irritable mood and temper outbursts separately, a series of logistic regressions with irritability, using the five-point Likert scale, sex, and their interaction as predictors, and each of the impairment indicators as outcome.

Given the multiple tests performed in the current study, we adjusted the false discovery rate (FDR) with Benjamini–Hochberg procedure setting FDR to 5%; therefore, inferences are protected from multiple testing using FDR correction. All analyses were performed in R version 4.4.1.

The results for irritability correlates during the childhood period, including examining sex effects based only on parent-report, are available in the supplemental information.

## Results

Overall, compared to adolescents without parent-reported irritability (without either irritable mood or temper outbursts), among those with parent-reported irritability there were more White participants, and fewer Asian or Black participants. Among those with irritability, especially among those with temper outbursts, there were fewer families owning a home and more families living in social housing and receiving benefits (Table 1, Table S1).

**Table 1:** Demographic characteristics of adolescent participants by informant, and presence of irritable mood and temper outbursts

	**Parent-report (ages 12 to 17)**				**Self-report (ages 12 to 17)**			
	**Irritable mood**		**Temper outbursts**		**Irritable mood**		**Temper outbursts**	
	**Absent** ***n* = 2105** **77%**	**Present** ***n* = 635** **23%**	**Absent** ***n* = 2336** **85%**	**Present** ***n* = 402** **15%**	**Absent** ***n* = 1830** **74%**	**Present** ***n* = 644** **26%**	**Absent** ***n* = 2095** **85%**	**Present** ***n* = 376** **15%**
**Age, *M* (*SD*)**	14.2 (1.6)	14.06 (1.6)	14.2 (1.6)	13.99 (1.5)	14.3 (1.7)	14.3 (1.6)	14.3 (1.7)	14.2 (1.7)
**Female, n (%)**	1064 (50.5)	314 (49.4)	1177 (50.4)	201 (50.0)	885 (48.4)	367 (57.0)	1045 (49.9)	204 (54.3)
**Ethnicity, *n* (%)**								
White British	1577 (75.0)	548 (86.3)	1775 (76.0)	348 (86.5)	1404 (76.7)	510 (79.2)	1612 (77.0)	299 (79.5)
Asian/Asian British	225 (10.7)	30 (4.7)	238 (10.2)	17 (4.2)	187 (10.2)	47 (7.3)	207 (9.9)	27 (7.2)
Black/African/Caribbean/Black British	96 (4.6)	11 (1.7)	102 (4.4)	5 (1.2)	75 (4.1)	21 (3.3)	82 (3.9)	14 (3.7)
Multi-ethnic	126 (6.0)	33 (5.2)	137 (5.9)	22 (5.5)	105 (5.7)	45 (7.0)	125 (6.0)	25 (6.6)
White other	80 (3.8)	13 (2.0)	83 (3.6)	10 (2.5)	105 (3.2)	21 (3.3)	68 (3.2)	11 (2.9)
**Housing tenure, *n* (%)**								
Owned	1379 (65.6)	421 (66.5)	1562 (67.0)	238 (59.4)	1251 (68.9)	395 (61.9)	1430 (68.7)	213 (57.6)
Privately rented	331 (15.7)	84 (13.3)	357 (15.3)	57 (14.2)	259 (14.3)	104 (16.3)	294 (14.1)	69 (18.6)
Social housing	393 (18.7)	128 (20.2)	414 (17.7)	106 (26.4)	306 (16.9)	139 (21.7)	357 (17.2)	88 (23.8)
**Benefits, *n* (%)**								
Parent/s income support	526 (28.1)	187 (32.7)	572 (27.5)	141 (39.4)	394 (25.5)	182 (32.3)	448 (25.3)	126 (38.4)
Any welfare benefits	609 (32.6)	337 (41.0)	676 (32.4)	168 (46.9)	467 (30.2)	209 (37.2)	532 (30.0)	1442 (43.3)

*Note 1*: Statistical comparisons of these demographic characteristics are reported in Table S1 in the supplement

*Note 2*: Demographic characteristics of child participants by informant, and presence of irritable mood and temper outbursts, is available in Table S2 in the supplement

### Age, sex, and informant effects on the prevalence of irritability

**Fig. 1:**
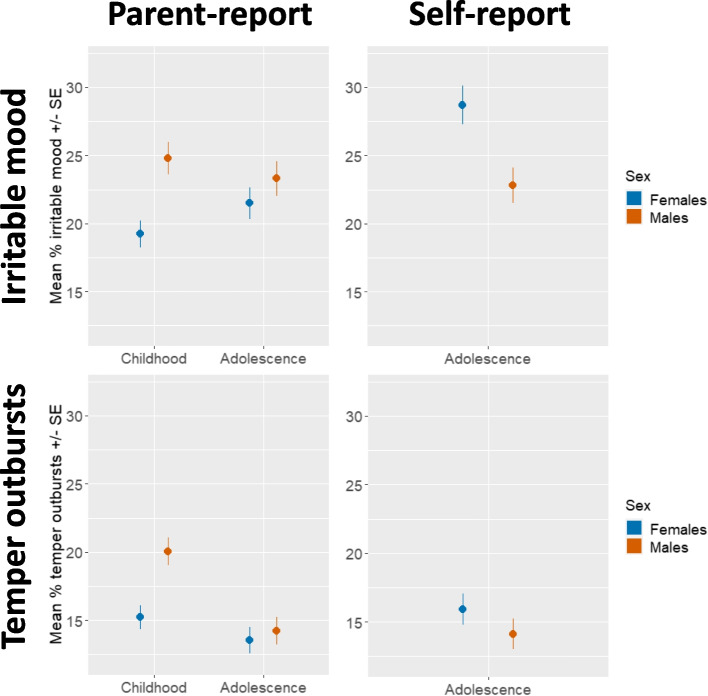
Percentage of frequent irritable mood and temper outbursts by sex and informant

Based on *parent*-report, both males and females showed similar prevalence of irritability in adolescence (Irritable mood: 23% males vs 22% females; Temper outbursts: 14% males vs 14% females). In childhood, however, the prevalence of irritability was higher in males (Irritable mood: 25% males vs 19% females; Temper outbursts: 20% males vs 15% females) (Table S3). This resulted in a sex by age interaction (Irritable mood: b = 0.12, SE = 0.05, *p* = 0.019; Temper outbursts: b = 0.10, SE = 0.05, *p* = 0.029, though the latter did not survive FDR correction) (Fig. 1, Table S4).

Based on *self*-report, adolescent females reported higher prevalence of irritable mood than males (23% males vs 29% females, b = 0.13, SE = 0.03, *p* < 0.001). Males and females did not differ on self-reported prevalence of temper outbursts (14% males vs 16% females, b = 0.04, SE = 0.04, *p* = 0.295) (Fig. 1, Table S3, Table S4). Results with ordinal linear regression treating irritability as an ordinal dependent variable replicated those using linear regression (Table S5).

### Sex and informant effects on the associations between irritability and psychopathological symptoms in adolescents

Higher frequency of irritability in adolescents was associated with higher levels of psychopathological symptoms and overall impairment. Table S6 presents all means and SE of SDQ subscales, by presence of irritability, sex, and informant in adolescents, whereas Table S7 presents the means and SE in childhood. However, there were specific sex and informant effects (Table 2).

**Table 2:** Results of linear regression by informant examining the association between irritable mood/temper outbursts, and their interaction with sex, and SDQ subscales scores in adolescence

**Parent report**	**Emotional problems**			**Hyperactivity problems**			**Conduct problems**			**Impact**		
	**B**	**SE**	***p*-value**	**B**	**SE**	***p*-value**	**B**	**SE**	***p*-value**	**B**	**SE**	***p*-value**
Irritable mood	0.85	0.08	< 0.001	1.10	0.08	< 0.001	0.95	0.07	< 0.001	0.94	0.08	< 0.001
Sex	0.38	0.15	0.013	−0.55	0.16	< 0.001	−0.11	0.10	0.284	−0.11	0.12	0.355
Irritable mood*Sex	0.21	0.12	0.074	−0.29	0.12	0.013	−0.07	0.09	0.440	0.00	0.12	0.991
Temper outbursts	0.91	0.08	< 0.001	1.26	0.09	< 0.001	1.07	0.07	< 0.001	0.98	0.09	< 0.001
Sex	0.47	0.13	< 0.001	−0.63	0.13	< 0.001	−0.13	0.08	0.091	−0.13	0.10	0.198
Temper outbursts*Sex	0.22	0.12	0.070	−0.24	0.11	0.032	−0.01	0.09	0.869	0.07	0.13	0.587
**Self-report**	**Emotional problems**			**Hyperactivity problems**			**Conduct problems**			**Impact**		
	**B**	**SE**	***p*-value**	**B**	**SE**	***p*-value**	**B**	**SE**	***p*-value**	**B**	**SE**	***p*-value**
Irritable mood	0.72	0.08	< 0.001	0.84	0.09	< 0.001	0.73	0.06	< 0.001	0.35	0.06	< 0.001
Sex	0.63	0.14	< 0.001	−0.64	0.17	< 0.001	−0.25	0.10	0.015	−0.15	0.10	0.142
Irritable mood*Sex	0.27	0.10	0.011	0.18	0.12	0.143	0.08	0.08	0.357	0.31	0.10	0.001
Temper outbursts	0.58	0.08	< 0.001	0.92	0.08	< 0.001	0.83	0.05	< 0.001	0.31	0.05	< 0.001
Sex	0.81	0.12	< 0.001	−0.47	0.13	< 0.001	−0.17	0.07	0.016	0.01	0.06	0.933
Temper outbursts*Sex	0.25	0.11	0.022	0.15	0.11	0.175	0.09	0.08	0.254	0.31	0.08	< 0.001

Results of linear regression examining the association between irritable mood/temper outbursts, and their interaction (denoted by an asterisk *) with sex, and SDQ subscales scores in childhood are in Table S7 in the supplement

**Fig. 2:**
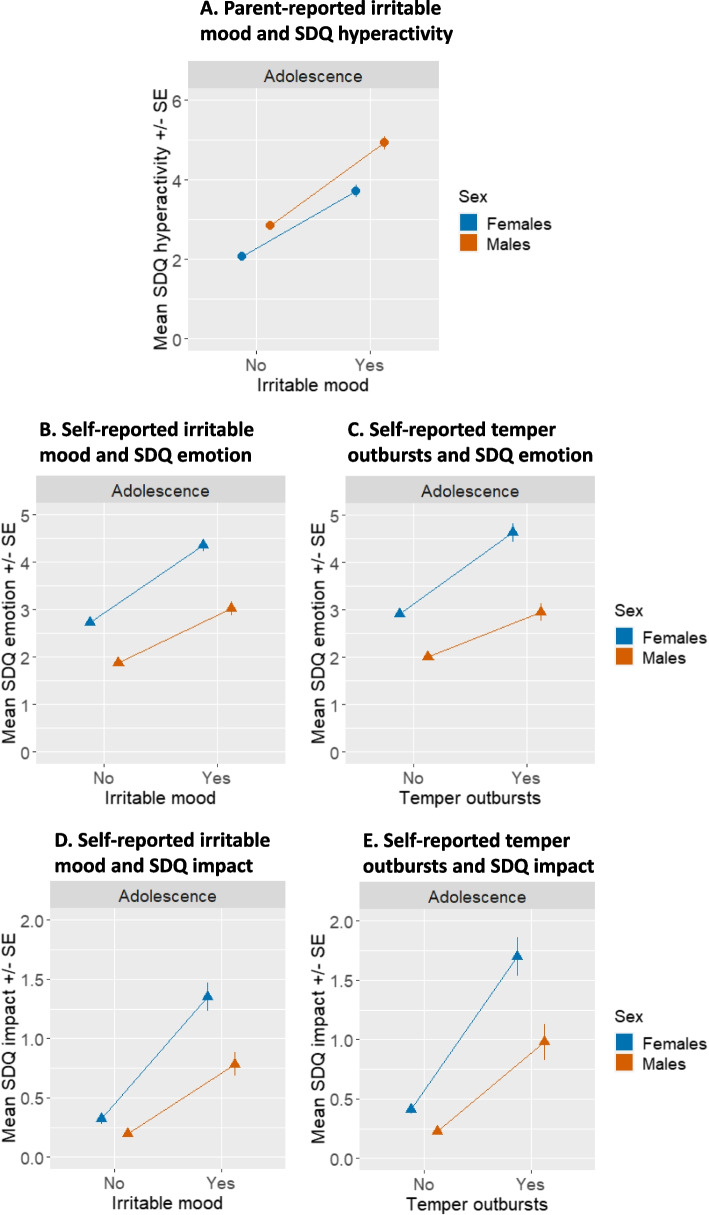
Mean of SDQ subscales scores (SDQ hyperactivity, panel **A**; SDQ emotion, panels **B** and **C**; SDQ impact, panels **D** and **E**) by presence of irritable mood (panels **A**, **B**, and **D**) or temper outbursts (panels **C** and **E**) as reported by parents (panel **A**) or adolescents (panels **B**, **C**, **D**, and **E**)

Based on parent-report, higher frequency of irritable mood was associated with higher levels of hyperactivity problems more strongly in males than females (Fig. 2A, Table 2; b = −0.29, SE = 0.12, *p* = 0.013). Interestingly, effect sizes were in the opposite direction for self-reported irritability, though the interaction was not significant (Figure S2, Table 2). However, based on self-report, in females vs. males, higher frequency of irritable mood and temper outbursts were more associated with emotional problems (Fig. 2B, irritable mood, b = 0.27, SE = 0.10, *p* = 0.011; Fig. 2C, temper outbursts: b = 0.25, SE = 0.11, *p* = 0.022) and overall impairment (Fig. 2D, irritable mood, b = 0.31, SE = 0.10, *p* = 0.001; Fig. 2E, temper outbursts: b = 0.31, SE = 0.08,

*p* < 0.001) (Table 2). Across informant associations are available in Table S8, whereas Table S9 presents associations between irritability and SDQ subscales in childhood.

### Sex and informant effects on the associations between irritability and psychiatric disorders in adolescents

Overall, prevalence of any internalizing disorder was higher in adolescent females than males, and prevalence of any externalizing disorder was higher in males than females. As expected, adolescents with irritability had a higher prevalence of psychiatric disorders than adolescents without irritability (Table S10; see Table S11 for childhood prevalences).

**Fig. 3:**
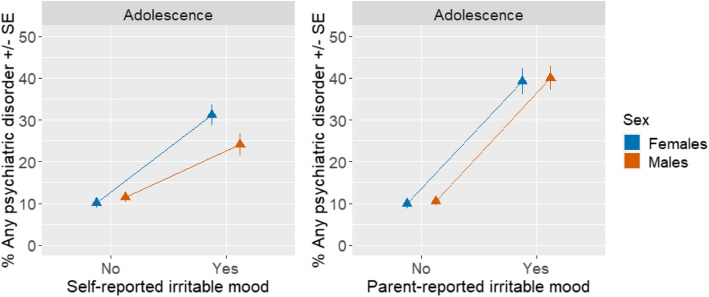
Percentage of any psychiatric disorder by self- and parent-reported irritable mood and sex in adolescence

In general, the sex ratio of psychiatric disorders was similar when comparing adolescents with and without irritability. However, the increase in prevalence of any psychiatric disorder associated with self-reported irritability was more prominent in adolescent females than males. Specifically, the prevalence of any psychiatric disorder in adolescents without self-reported irritable mood was 11.5% for males and 10.1% for females (sex ratio 1.1:1), versus 24.1% in males and 31.2% in females with self-reported irritable mood (sex ratio 0.8:1) (Table S10). This was evidenced by an interaction of frequency of irritable mood by sex (b = 0.35, SE = 0.15, *p* = 0.020) (Fig. 3, Table S12). This effect was not seen with parent-reported irritability. Associations between irritability and psychiatric diagnoses in childhood by sex are reported in Table S13.

Irritable mood and temper outbursts in adolescence were associated with self-harming behaviors, school exclusions, and contact with mental health services, with no apparent sex or informant effects (Table S14). Similar results were seen in childhood (Table S15).

## Discussion

Using a large population-based cohort of children and adolescents, we found that informant, age, and sex influence the reporting of irritability. Parents reported similar levels of irritability (i.e., irritable mood and temper outbursts) in adolescent males and females, which contrasted with higher parent-reported irritability in males during childhood. In contrast, adolescent females self-reported more irritable mood than did adolescent males. This self-reported irritability in adolescent females was associated with greater emotional problems and overall impairment compared to males. In contrast, parent-reported irritability was more associated with hyperactivity problems in males than females. The relative increase in psychiatric disorders associated with irritability in adolescence was greater in females than males.

As noted above, parents reported higher levels of both irritable mood and temper outbursts in males vs. females during childhood but similar levels during adolescence. Indeed, parent-reported temper outbursts decreased in adolescence compared to childhood for everyone. This decrease could be attributed to the development of self-regulation skills during adolescence, associated with prefrontal cortical maturation and improved brain function [37]. In contrast, moving from childhood to adolescence, parent-reported irritable mood showed a stable pattern in males but an increase in females, with the net result being similar levels of parent-reported irritable mood in adolescent males and females. These findings support previous parent-report community studies [14].

While parent report showed an increase in irritable mood in females from childhood to adolescence, the highest levels of irritable mood was reported by adolescent females themselves. Thus, irritable mood self-reported by adolescent females was significantly higher than both adolescent males’ self-reports and parent reports. Data from non-referred samples using self-reported irritability during adolescence have also found higher levels of irritability in females than males [17]; our study further shows that the full extent of female adolescents’ irritable mood might go unnoticed by parents.

In considering why irritable mood, but not temper outbursts, is more common by self- than parent-report in adolescent females, the answer may lie in the expression of irritable mood as opposed to temper outbursts. Temper outbursts are observable and easily noticeable to external informants such as parents, as they are usually displayed by verbal or physical aggression. In contrast, irritable mood entails the experience of affect and may not be necessarily expressed or noticed externally even if deeply felt [38]. As reported by both parents and adolescents themselves, increased irritable mood occurs in females, but not males, during adolescence, possibly reflecting the higher incidence of psychopathology in females in this developmental period, since irritable mood is a core feature in depression, generalized anxiety, and personality disorders characterized by emotion dysregulation [39]. Notably, the association between self-reported irritable mood and other emotional symptoms (e.g., somatic complaints, worries, unhappiness, and anxiety) was stronger among adolescent females than males, and adolescent females with irritability experienced higher levels of overall impairment than males. In contrast, compared to females, adolescent males with parent-reported irritable mood exhibited increased levels of hyperactivity symptoms. In childhood, temper outbursts were also more associated with hyperactivity and conduct problems in males than females. These findings showing distinct sex associations with psychopathological symptoms are consistent with previous reports suggesting that irritability in adolescence might be more related to internalizing symptoms and female sex [27], whereas irritability in childhood might be more related to externalizing symptoms and male sex [13, 14]. Distinct correlates with irritable mood and temper outbursts also support the distinction of these two expressions of irritability pathophysiologically.

Self-reported irritable mood was associated with a higher risk of psychiatric disorders in females with irritability than in males with irritability. Perhaps surprisingly, this effect was more evident for externalizing (B = 0.36) than internalizing disorders (B = 0.15). In childhood, both parent-reported irritable mood and temper outbursts were also associated with a higher risk of any externalizing disorder in females than males. It is important to note, though, that the prevalence of externalizing disorders was higher in males at any age and increased for both sexes with irritability; however, the interactions revealed that the relative increase compared to those without irritability was higher in females.

In the DAWBA, criteria for externalizing disorders primarily rely on parental accounts of behaviors. Our results suggest, therefore, that parents might consider irritability in females, in contrast to males, a more significant factor when providing information for clinicians screening externalizing disorders. The reasons for behind this phenomenon are unclear. It could be influenced by gender roles and social norms, leading parents to perceive female irritability as something extremely unexpected, thereby elevating the likelihood of an externalizing disorder diagnosis [40]. Alternatively, some of this irritability in females may occur within the context of internalizing disorders, more common in adolescent females than males. In this context, irritability might heighten the probability of coexisting externalizing disorders and be a symptom more easily identified by parents, as it manifests more overtly than internalizing psychopathology such as sadness or anxiety. Future research should clarify the factors that contribute to the relative increased risk of externalizing disorders in females with irritability.

Irritability was associated with self-harming behaviors, school exclusions, and utilization of mental health services, irrespective of sex, age, or informant source. This suggests that irritability may be a robust factor in identifying these clinically meaningful impairment outcomes at a population level regardless of when it is reported and who is reporting it.

While our study benefits from a large community-based sample and a multi-informant approach, it is not without limitations. First, the cross-sectional design of the study prevents us from establishing a causal relationship between irritability and other psychopathological symptoms. Therefore, it is unclear whether irritability is a risk factor for other psychopathological symptoms such as emotional psychopathology in females or ADHD symptoms in males, or alternatively, irritability is another manifestation of this psychopathology. Similarly, we report prevalence of psychopathology at a single time point, so we cannot demonstrate that, for example, irritable mood increases from childhood to adolescence in females, although this is likely based on previous reports from longitudinal studies [14]. Second, multi-informant assessments were available in the adolescent period, but only parent-reports were available for younger children. Third, while survey weights minimized bias from known factors influencing non-response, unmeasured confounders may still have had an impact. However, the strong agreement between unweighted and weighted estimators (e.g., Table S2) suggests that this is unlikely to significantly affect our findings. Lastly, one limitation of this study is the inherent complexity of defining and measuring irritability. While we adopt a working definition of irritability as a proneness to anger that allows us to approach the study of the construct cautiously, we recognize that irritability is conceptualized in multiple ways across the literature (Toohey & DiGiuseppe, 2017; Barata et al., 2016). The boundaries between irritability and related constructs such as anger, frustration, agitation, and aggression has been and remain an area of ongoing research (Stringaris et al., 2018; Toohey & DiGiuseppe, 2017; Vidal-Ribas et al., 2016). For example, our item assessing irritability includes both"irritable mood"and"angry mood", which reflects the conceptual overlap between these phenomenologically distinct constructs and acknowledges that the general public might use both interchangeably. Moreover, our assessment relied on a single question each for irritable mood and temper outbursts, which focused solely on their frequency. While a lowered threshold for anger increases its frequency, in line with our definition of irritability, a high frequency of anger does not necessarily imply a reduced threshold for it (Toohey & DiGiuseppe, 2017). A more comprehensive approach would consider additional aspects such as threshold, duration, onset, situational context, and specific behavioral expressions of irritability. Addressing these nuances in future studies will be crucial for advancing the conceptualization and measurement of irritability, as emphasized in our recent review (Leibenluft et al., 2024).

## Conclusion

Despite these limitations, our results have several implications for research design and clinical practice. First, they highlight the importance of considering adolescents’ perspectives when assessing their mental health problems, particularly regarding their experience of affect that might not be observable by external informants such as caregivers. Second, the identification of irritability by adolescent themselves, especially in females, should make us wary of the co-occurrence of other emotional problems, such as depressive and anxiety symptoms, that may be sufficiently severe to meet criteria for an affective disorder. Lastly, the identification of irritability in females by themselves and their parents may serve as a potential indicator of the presence of an externalizing disorder. Taken together, our findings contribute to a better understanding of irritability from the perspective of parents and children of different ages and sexes and underscore the need for comprehensive assessments that consider these multiple factors in the measurement of irritability.

## Supplementary Information

Supplementary Material 1.

## Data Availability

Availability of data and materials: Approved researchers seeking to undertake further secondary analysis of data from the MHCYP surveys will be able to access the data from the UK Data Service under Special User Licence. Applicants will need to apply for permission to use the data via NHS England Data Access Request Service (https://digital.nhs.uk/services/data-access-request-service-dars). Once approval has been granted, the data can be downloaded from the UK Data Service (https://beta.ukdataservice.ac.uk/datacatalogue/studies/study?id=8467). You can find out more about access at the UK Data Service webpage (https://ukdataservice.ac.uk/) and the NHS England Population health surveys webpage (https://digital.nhs.uk/services/data-access-request-service-dars/dars-products-and-services/population-health-surveys).

## Abbreviations

DAWBA: Development and Well-Being Assessment
DMDD: Disruptive mood dysregulation disorder
SDQ: Strengths and Difficulties Questionnaire
