# Supplementary material for: The hidden burden: self-reported irritability in adolescent girls signals higher psychiatric risk
Source: BMC Public Health. 2025 May 19;25:1832. doi: 10.1186/s12889-025-23076-6 (PMC12087224; doi:10.1186/s12889-025-23076-6)
Supplement: Supplementary file 1 — Supplementary Material 1. [file 12889_2025_23076_MOESM1_ESM.docx]

**The hidden burden: self-reported irritability in adolescent girls signals higher psychiatric risk**

Pablo Vidal-Ribas, PhD, Georgina Krebs, PhD, DClinPsy, Jamilah Silver, M.A., Wan-Ling Tseng, PhD, Tamsin Ford, PhD, Ellen Leibenluft, MD, Argyris Stringaris, MD, PhD, FRCPsych

**Supplemental Information**


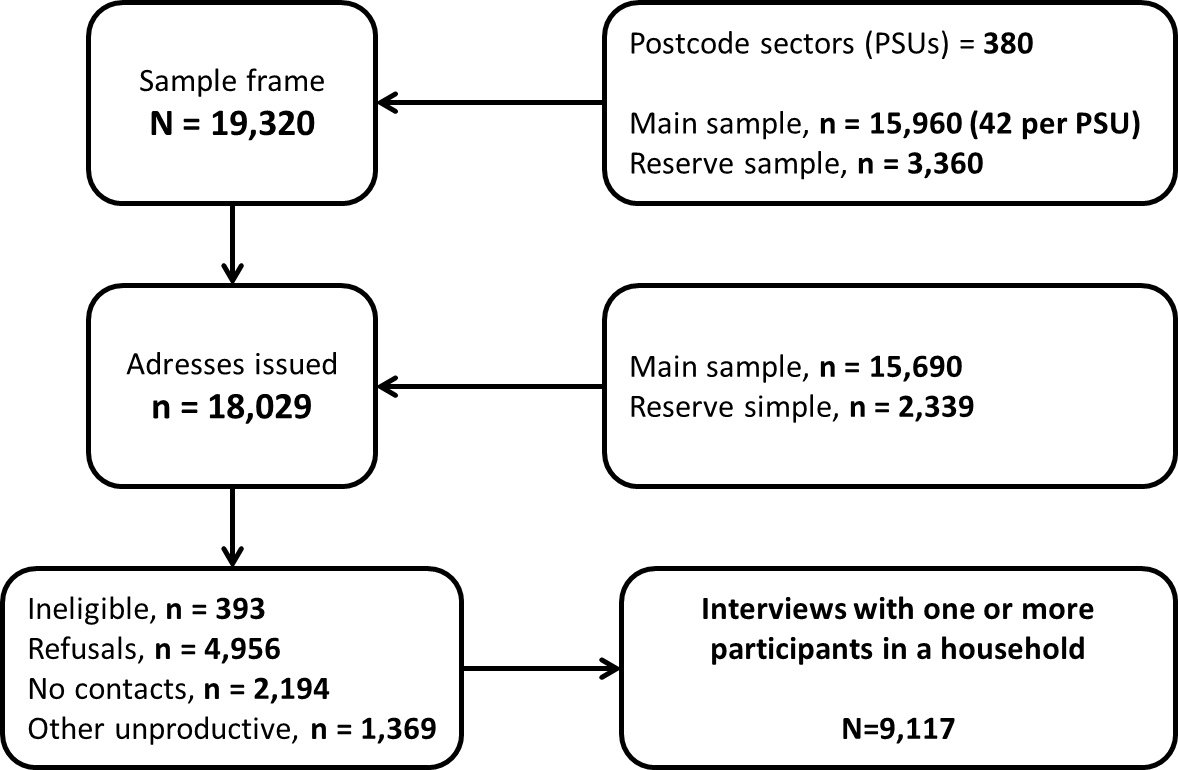


**Figure S1. Participant recruitment to the Child and Young People´s Mental Health Survey 2017. Adapted from Ford et al., 2020.**

**Data weighting procedure**

Briefly, the weighting procedures outlined in the Mental Health of Children and Young People (MHCYP) 2017 Survey Design and Methods report (pages 48-51) involved the following key steps to ensure that survey results were representative of the 2-19-year-old population in England, effectively addressing both sampling and non-response biases:

1. **Design Weights**:
   - **Main and Reserve Samples**: The survey accounted for the probability of selection at two levels: the Primary Sampling Units (PSUs) and individual children/young people. Design weights were calculated separately for the main and reserve samples, and then combined. PSU selection weights accounted for differential probabilities of postcode sectors, while individual selection weights accounted for child age groups within those sectors​​.
2. **Non-Response Weighting**:
   - To mitigate the effects of non-response bias, logistic regression models were applied on the response indicator variable using the age used for sample selection (grouped), region (at selection) and Index of Multiple Deprivation (IMD), and the source of the data (main or reserve sample) as additional variables. These were the variables that were found to be significant predictors of response. Other variables were tested (e.g. ethnic group, urban/rural indicator, and tenure) but not used within the final model as they were not significant. The non-response adjustment factors were equal to the inverse of the predicted probabilities from the fitted model. They were applied to combined design weights to obtain the pre-calibration weights.
3. **Calibration**:
   - Calibration weighting of the pre-calibration weights was used to improve precision and reduce the bias of population estimates produced by the sample, by adjusting the sample’s design weights. The calibration adjusted the design weights produced, to reproduce known population totals at specified levels of aggregation. ONS population figures (which were calibrated to the month of August 2017) were used to calibrate by age group (2 to 4, 5 to 9,10 to 15,16,17 to 19), sex and region for those aged 2 to 19.
4. **Teacher Adjustment Factors**:
   - For children aged 5-16, a teacher non-response adjustment was included to account for the differential availability of teacher data, ensuring comparability across age groups. A single teacher factor was calculated for each mental disorder type and applied across all 5 to 16 year olds. The 2017 survey implemented a small improvement to calculate adjustments on weighted data; the new approach reduced the bias in the factors as they were representative of the whole population not purely the unweighted survey data.

**Table S1. Statistical comparison of demographic characteristics of adolescent participants by presence of irritable mood and temper outbursts reported by parents.**

|  | **Parent-report (ages 12 to 17)** | | | | | |
| --- | --- | --- | --- | --- | --- | --- |
|  | **Irritable mood** | | | **Temper outbursts** | | |
|  | **Absent**  ***n* = 2105**  **77%** | **Present**  ***n* = 635**  **23%** | **Statistical comparison** | **Absent**  ***n* = 2336**  **85%** | **Present**  ***n* = 402**  **15%** | **Statistical comparison** |
| **Female, n (%)** | 1064 (50.5) | 314 (49.4) | χ^2^ = 0.19; *p* = .660 | 1177 (50.4) | 201 (50.0) | χ^2^ = .01; *p* = .920 |
| **Ethnicity, *n* (%)** |  |  |  |  |  |  |
| White British | 1577 (75.0) | 548 (86.3) | χ^2^ = 41.72; *p* <.001 | 1775 (76.0) | 348 (86.5) | χ^2^ = 27.82; *p* < .001 |
| Asian /Asian British | 225 (10.7) | 30 (4.7) |  | 238 (10.2) | 17 (4.2) |  |
| Black/African/Caribbean/Black British | 96 (4.6) | 11 (1.7) |  | 102 (4.4) | 5 (1.2) |  |
| Multi-ethnic | 126 (6.0) | 33 (5.2) |  | 137 (5.9) | 22 (5.5) |  |
| White other | 80 (3.8) | 13 (2.0) |  | 83 (3.6) | 10 (2.5) |  |
| **Housing tenure, *n* (%)** |  |  |  |  |  |  |
| Owned | 1379 (65.6) | 421 (66.5) | χ^2^ = 2.62; *p* = .270 | 1562 (67.0) | 238 (59.4) | χ^2^ = 16.9; *p* < .001 |
| Privately rented | 331 (15.7) | 84 (13.3) |  | 357 (15.3) | 57 (14.2) |  |
| Social housing | 393 (18.7) | 128 (20.2) |  | 414 (17.7) | 106 (26.4) |  |
| **Benefits, *n* (%)** |  |  |  |  |  |  |
| Parent/s income support | 526 (28.1) | 187 (32.7) | χ^2^ = 4.27; *p* = 0.040 | 572 (27.5) | 141 (39.4) | χ^2^ = 20.28; *p* < .001 |
| Any welfare benefits | 609 (32.6) | 337 (41.0) | χ^2^ = 13.33; *p* < .001 | 676 (32.4) | 168 (46.9) | χ^2^ = 27.93; *p* < .001 |
|  | **Self-report (ages 12 to 17)** | | | | | |
|  | **Irritable mood** | | | **Temper outbursts** | | |
|  | **Absent**  ***n* = 1830**  **74%** | **Present**  ***n* = 644**  **26%** | **Statistical comparison** | **Absent**  ***n* = 2095**  **85%** | **Present**  ***n* = 376**  **15%** | **Statistical comparison** |
| **Female, n (%)** | 885 (48.4) | 367 (57.0) | χ^2^ = 13.84; *p* < .001 | 1045 (49.9) | 204 (54.3) | χ^2^ = 2.27; *p* = .130 |
| **Ethnicity, *n* (%)** |  |  |  |  |  |  |
| White British | 1404 (76.7) | 510 (79.2) | χ^2^ = 6.77; *p* = .150 | 1612 (77.0) | 299 (79.5) | χ^2^ = 3.10; *p* = .540 |
| Asian /Asian British | 187 (10.2) | 47 (7.3) |  | 207 (9.9) | 27 (7.2) |  |
| Black/African/Caribbean/Black British | 75 (4.1) | 21 (3.3) |  | 82 (3.9) | 14 (3.7) |  |
| Multi-ethnic | 105 (5.7) | 45 (7.0) |  | 125 (6.0) | 25 (6.6) |  |
| White other | 105 (3.2) | 21 (3.3) |  | 68 (3.2) | 11 (2.9) |  |
| **Housing tenure, *n* (%)** |  |  |  |  |  |  |
| Owned | 1251 (68.9) | 395 (61.9) | χ^2^ = 11.1; *p* < .010 | 1430 (68.7) | 213 (57.6) | χ^2^ = 17.76; *p* < .001 |
| Privately rented | 259 (14.3) | 104 (16.3) |  | 294 (14.1) | 69 (18.6) |  |
| Social housing | 306 (16.9) | 139 (21.7) |  | 357 (17.2) | 88 (23.8) |  |
| **Benefits, *n* (%)** |  |  |  |  |  |  |
| Parent/s income support | 394 (25.5) | 182 (32.3) | χ^2^ = 9.38; *p* < .010 | 448 (25.3) | 126 (38.4) | χ^2^ = 23.50; *p* < .001 |
| Any welfare benefits | 467 (30.2) | 209 (37.2) | χ^2^ = 8.74; *p* < .010 | 532 (30.0) | 1442 (43.3) | χ^2^ = 21.89; *p* < .001 |

**Table S2. Demographic characteristics of children participants by presence of irritable mood and temper outbursts based on parent-report.**

|  | **Parent-report (ages 5 to 11)** | | | |
| --- | --- | --- | --- | --- |
|  | **Irritable mood** | | **Temper outbursts** | |
|  | **Absent**  ***n* = 3217**  **78%** | **Present**  ***n* = 924**  **22%** | **Absent**  ***n* = 3390**  **82%** | **Present**  ***n* = 750**  **18%** |
| **Age, *M* (*SD*)** | 7.9 (2.0) | 8.0 (2.0) | 7.9 (2.0) | 7.9 (2.0) |
| **Female, n (%)** | 1646 (51.2) | 401 (43.4) | 1727 (50.9) | 320 (42.7) |
| **Ethnicity, *n* (%)** |  |  |  |  |
| White British | 2311 (71.9) | 776 (84.0) | 2455 (72.4) | 631 (84.1) |
| Asian /Asian British | 380 (11.8) | 56 (6.1) | 396 (11.7) | 40 (5.3) |
| Black/African/Caribbean/Black British | 159 (4.9) | 19 (2.1) | 165 (4.9) | 13 (1.7) |
| Multi-ethnic | 231 (7.2) | 46 (5.0) | 228 (6.7) | 49 (6.5) |
| White other | 135 (4.2) | 27 (2.9) | 145 (4.3) | 17 (2.3) |
| **Housing tenure, *n* (%)** |  |  |  |  |
| Owned | 1889 (58.8) | 501 (54.3) | 1562 (67.0) | 238 (59.4) |
| Privately rented | 670 (20.9) | 170 (18.4) | 357 (15.3) | 57 (14.2) |
| Social housing | 654 (20.4) | 252 (27.3) | 414 (17.7) | 106 (26.4) |
| **Benefits, *n* (%)** |  |  |  |  |
| Parent/s income support | 929 (31.1) | 324 (3.8) | 963 (30.7) | 289 (41.7) |
| Any welfare benefits | 1047 (35.1) | 490 (57.6) | 1073 (34.2) | 334 (48.0) |

**Table S3. Percentage of participants with irritable mood and temper outbursts, by sex, informant and age period.**

|  |  |  |  |
| --- | --- | --- | --- |
|  |  | **Irritable mood** | |
|  |  | **Weighted** | **Observed (unweighted)** |
| **Informant** |  | ***% (95%CI)*** | ***n (%)*** |
| **Parent** |  |  |  |
| 5-11 years | Males | 24.8 (22.5, 27.1) | 522 (25.0) |
|  | Females | 19.2 (17.3, 21.2) | 400 (19.6) |
| 12-17 years | Males | 23.3 (20.8, 25.8) | 320 (23.5) |
|  | Females | 21.5 (19.2, 23.8) | 313 (22.8) |
| **Child** |  |  |  |
| 12-17 years | Males | 22.8 (20.3, 25.4) | 279 (22.8) |
|  | Females | 28.7 (26.0, 31.4) | 364 (29.1) |
|  |  |  |  |
|  |  | **Temper outbursts** | |
|  |  | **Weighted** | **Observed (unweighted)** |
| **Informant** |  | ***% (95%CI)*** | ***n (%)*** |
| **Parent** |  |  |  |
| 5-11 years | Males | 20.0 (18.0, 22.0) | 431 (20.6) |
|  | Females | 15.2 (13.5, 17.0) | 319 (15.6) |
| 12-17 years | Males | 14.2 (12.2, 16.2) | 202 (14.8) |
|  | Females | 13.6 (11.7, 15.4) | 200 (14.6) |
| **Child** |  |  |  |
| 12-17 years | Males | 14.1 (11.9, 16.3) | 173 (14.2) |
|  | Females | 15.9 (13.7, 18.2) | 202 (16.2) |
|  |  |  |  |
|  |  |  |  |

**Table S4. Results of linear regression examining the effects of sex and age on irritable mood and temper outbursts by informant.**

|  |  |  |  |  |  |  |
| --- | --- | --- | --- | --- | --- | --- |
|  | **Irritable mood** | | | **Temper outbursts** | | |
| **Parent report** | **B** | **SE** | ***p*-value** | **B** | **SE** | ***p*-value** |
| **Sex** | -0.14 | 0.03 | <0.001 | -0.17 | 0.03 | <0.001 |
| **Age** | -0.02 | 0.04 | 0.578 | -0.13 | 0.03 | <0.001 |
| **Sex*Age** | 0.12 | 0.05 | 0.019 | 0.10 | 0.05 | 0.029 |
| **Self-report** |  |  |  |  |  |  |
| **Sex** | 0.13 | 0.03 | 0.001 | 0.02 | 0.08 | 0.804 |

**Table S5. Results of ordinal linear regression examining the effects of sex and age on irritable mood and temper outbursts by informant.**

|  |  |  |  |  |  |  |
| --- | --- | --- | --- | --- | --- | --- |
|  | **Irritable mood** | | | **Temper outbursts** | | |
| **Parent report** | **B** | **SE** | ***p*-value** | **B** | **SE** | ***p*-value** |
| **Sex** | -0.31 | 0.07 | <0.001 | -0.37 | 0.06 | <0.001 |
| **Age** | -0.05 | 0.08 | 0.485 | -0.31 | 0.07 | <0.001 |
| **Sex*Age** | 0.29 | 0.11 | 0.006 | 0.25 | 0.10 | 0.014 |
| **Self-report** |  |  |  |  |  |  |
| **Sex** | 0.25 | 0.07 | <0.001 | 0.04 | 0.04 | 0.295 |

**Table S6. Mean scores of SDQ subscales for participants with and without irritable mood and temper outbursts, by sex and informant in adolescence.**

|  |  |  |  |  |  |  |  |  |  |
| --- | --- | --- | --- | --- | --- | --- | --- | --- | --- |
|  |  | **Emotional problems** | | **Hyperactivity problems** | | **Conduct problems** | | **Impact** | |
|  |  | **Without irritability** | **With irritability** | **Without irritability** | **With irritability** | **Without irritability** | **With irritability** | **Without irritability** | **With irritability** |
|  |  | ***M (SE)*** | ***M (SE)*** | ***M (SE)*** | ***M (SE)*** | ***M (SE)*** | ***M (SE)*** | ***M (SE)*** | ***M (SE)*** |
| **Irritable mood by parent report** | | | | | | | | | |
| **Adolescence** | **Males** | 1.65 (0.07) | 3.28 (0.17) | 2.86 (0.08) | 4.94 (0.17) | 0.97 (0.05) | 2.76 (0.14) | 0.49 (0.05) | 2.19 (0.18) |
|  | **Females** | 2.18 (0.07) | 4.23 (0.16) | 2.08 (0.07) | 3.72 (0.16) | 0.82 (0.04) | 2.48 (0.13) | 0.37 (0.04) | 2.15 (0.17) |
| **Temper outbursts by parent report** | | | | | | | | | |
| **Adolescence** | **Males** | 1.71 (0.07) | 3.95 (0.2) | 2.95 (0.08) | 5.75 (0.23) | 1.04 (0.05) | 3.48 (0.17) | 0.54 (0.05) | 2.94 (0.23) |
|  | **Females** | 2.3 (0.07) | 4.64 (0.21) | 2.13 (0.07) | 4.38 (0.21) | 0.84 (0.04) | 3.3 (0.16) | 0.41 (0.04) | 2.9 (0.23) |
| **Irritable mood by self-report** | | | | | | | | | |
| **Adolescence** | **Males** | 1.88 (0.06) | 3.03 (0.14) | 3.37 (0.08) | 4.66 (0.16) | 1.43 (0.06) | 2.56 (0.1) | 0.2 (0.03) | 0.78 (0.1) |
|  | **Females** | 2.73 (0.08) | 4.36 (0.14) | 2.88 (0.08) | 4.53 (0.13) | 1.27 (0.05) | 2.5 (0.11) | 0.32 (0.04) | 1.35 (0.12) |
| **Temper outbursts by self-report** | | | | | | | | | |
| **Adolescence** | **Males** | 2.01 (0.06) | 2.96 (0.19) | 3.42 (0.07) | 5.12 (0.18) | 1.47 (0.05) | 3 (0.13) | 0.22 (0.03) | 0.98 (0.15) |
|  | **Females** | 2.92 (0.07) | 4.63 (0.19) | 3.03 (0.08) | 5.05 (0.17) | 1.33 (0.05) | 3.14 (0.15) | 0.41 (0.04) | 1.7 (0.16) |

**
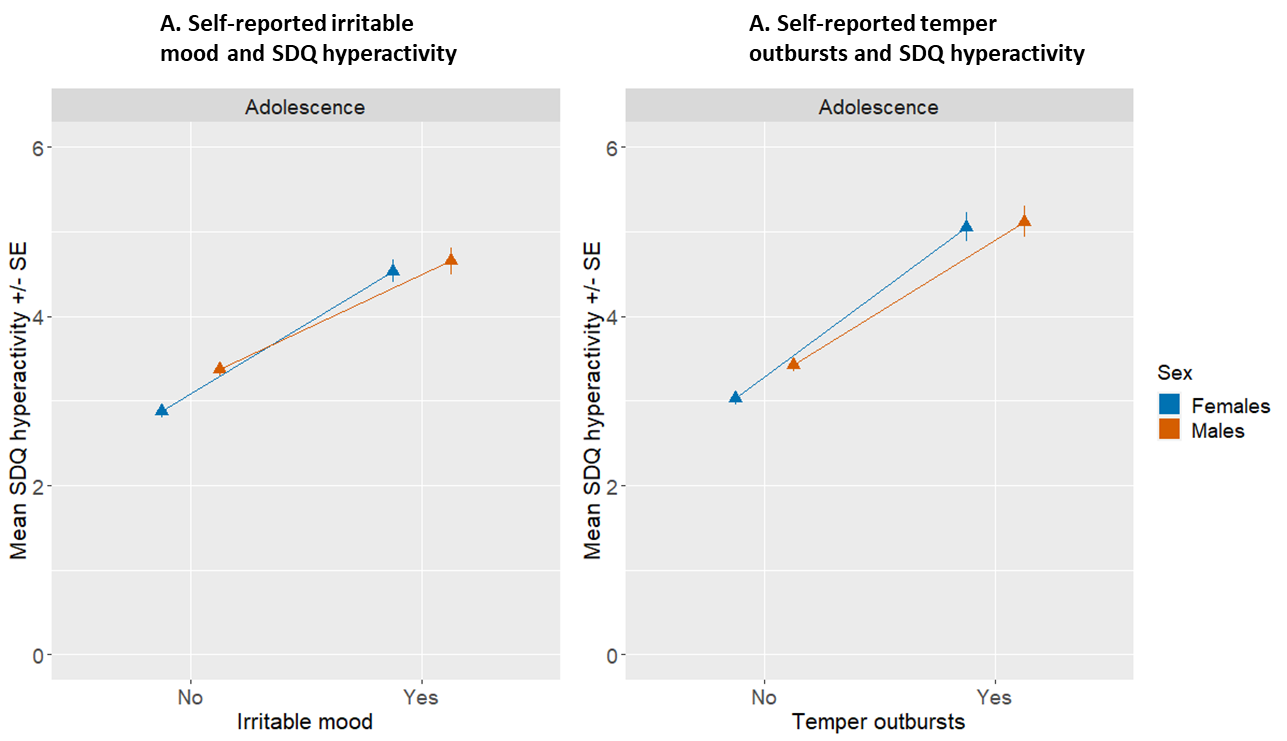
**

**Figure S2.** Mean of SDQ hyperactivity subscales scores by presence of irritable mood (panels A) or temper outbursts (panels B) as reported by adolescents.

In childhood, temper outbursts as reported by parents were more associated with hyperactivity (b=-0.32, SE=0.10, *p*=0.002) and conduct problems (b=-0.16, SE=0.07, *p*=0.025) in males than females (**Table S7, Table S9)**

**Table S7. Mean scores of SDQ subscales for participants with and without irritable mood and temper outbursts, by sex in childhood.**

|  |  |  |  |  |  |  |  |  |  |
| --- | --- | --- | --- | --- | --- | --- | --- | --- | --- |
|  |  | **Emotional problems** | | **Hyperactivity problems** | | **Conduct problems** | | **Impact** | |
|  |  | **Without irritability** | **With irritability** | **Without irritability** | **With irritability** | **Without irritability** | **With irritability** | **Without irritability** | **With irritability** |
|  |  | ***M (SE)*** | ***M (SE)*** | ***M (SE)*** | ***M (SE)*** | ***M (SE)*** | ***M (SE)*** | ***M (SE)*** | ***M (SE)*** |
| **Irritable mood by parent report** | | | | | | | | | |
| **Childhood** | **Males** | 1.67 (0.05) | 3.14 (0.12) | 3.24 (0.07) | 5.41 (0.14) | 1.14 (0.04) | 3.03 (0.11) | 0.41 (0.04) | 1.94 (0.13) |
|  | **Females** | 1.73 (0.05) | 3.3 (0.14) | 2.4 (0.06) | 4.26 (0.16) | 0.92 (0.03) | 2.51 (0.12) | 0.23 (0.03) | 1.45 (0.14) |
| **Temper outbursts by parent report** | | | | | | | | | |
| **Childhood** | **Males** | 1.7 (0.05) | 3.35 (0.14) | 3.23 (0.07) | 5.98 (0.16) | 1.15 (0.04) | 3.46 (0.12) | 0.4 (0.04) | 2.31 (0.16) |
|  | **Females** | 1.8 (0.05) | 3.33 (0.16) | 2.46 (0.06) | 4.43 (0.19) | 0.92 (0.03) | 2.91 (0.13) | 0.23 (0.02) | 1.75 (0.18) |

**Table S8. Results of linear regression by informant examining the association between irritable mood/temper outbursts, and their interaction with sex, and SDQ subscales scores across informants in adolescence.**

|  |  |  |  |  |  |  |  |  |  |  |  |  |
| --- | --- | --- | --- | --- | --- | --- | --- | --- | --- | --- | --- | --- |
| **Parent report of irritability** | **Emotional problems** | | | **Hyperactivity problems** | | | **Conduct problems** | | | **Impact** | | |
|  | **B** | **SE** | ***p*-value** | **B** | **SE** | ***p*-value** | **B** | **SE** | ***p*-value** | **B** | **SE** | ***p*-value** |
| **Irritable mood** | 0.37 | 0.07 | <0.001 | 0.66 | 0.08 | <0.001 | 0.59 | 0.06 | <0.001 | 0.19 | 0.04 | <0.001 |
| **Sex** | 0.76 | 0.14 | <0.001 | -0.31 | 0.16 | 0.062 | -0.11 | 0.10 | 0.294 | 0.03 | 0.08 | 0.742 |
| **Irritable mood*Sex** | 0.22 | 0.11 | 0.044 | -0.07 | 0.12 | 0.594 | 0.01 | 0.09 | 0.926 | 0.21 | 0.08 | 0.008 |
|  |  |  |  |  |  |  |  |  |  |  |  |  |
| **Temper outbursts** | 0.45 | 0.08 | <0.001 | 0.79 | 0.09 | <0.001 | 0.71 | 0.06 | <0.001 | 0.22 | 0.06 | <0.001 |
| **Sex** | 0.90 | 0.12 | <0.001 | -0.34 | 0.14 | 0.017 | -0.10 | 0.09 | 0.231 | 0.11 | 0.07 | 0.103 |
| **Temper outbursts*Sex** | 0.14 | 0.12 | 0.244 | -0.03 | 0.12 | 0.791 | 0.03 | 0.09 | 0.745 | 0.18 | 0.09 | 0.050 |
|  |  |  |  |  |  |  |  |  |  |  |  |  |
| **Self-report of irritability** | **Emotional problems** | | | **Hyperactivity problems** | | | **Conduct problems** | | | **Impact** | | |
|  | **B** | **SE** | ***p*-value** | **B** | **SE** | ***p*-value** | **B** | **SE** | ***p*-value** | **B** | **SE** | ***p*-value** |
| **Irritable mood** | 0.45 | 0.10 | <0.001 | 0.54 | 0.11 | <0.001 | 0.51 | 0.08 | <0.001 | 0.44 | 0.09 | <0.001 |
| **Sex** | 0.29 | 0.17 | 0.084 | -1.01 | 0.19 | <0.001 | -0.33 | 0.12 | 0.006 | -0.26 | 0.13 | 0.047 |
| **Irritable mood*Sex** | 0.27 | 0.13 | 0.046 | 0.08 | 0.14 | 0.584 | 0.08 | 0.10 | 0.419 | 0.15 | 0.13 | 0.242 |
|  |  |  |  |  |  |  |  |  |  |  |  |  |
| **Temper outbursts** | 0.49 | 0.09 | <0.001 | 0.83 | 0.09 | <0.001 | 0.72 | 0.07 | <0.001 | 0.48 | 0.09 | <0.001 |
| **Sex** | 0.44 | 0.13 | <0.001 | -0.81 | 0.14 | <0.001 | -0.15 | 0.08 | 0.061 | -0.17 | 0.09 | 0.076 |
| **Temper outbursts*Sex** | 0.25 | 0.13 | 0.054 | -0.07 | 0.13 | 0.596 | -0.04 | 0.09 | 0.699 | 0.14 | 0.12 | 0.247 |

**Table S9. Results of linear regression by informant examining the association between irritable mood/temper outbursts, and their interaction with sex, and SDQ subscales scores in childhood.**

|  |  |  |  |  |  |  |  |  |  |  |  |  |
| --- | --- | --- | --- | --- | --- | --- | --- | --- | --- | --- | --- | --- |
| **Parent report** | **Emotional problems** | | | **Hyperactivity problems** | | | **Conduct problems** | | | **Impact** | | |
|  | **B** | **SE** | ***p*-value** | **B** | **SE** | ***p*-value** | **B** | **SE** | ***p*-value** | **B** | **SE** | ***p*-value** |
| **Irritable mood** | 0.76 | 0.06 | <0,001 | 1.15 | 0.07 | <0,001 | 1.01 | 0.05 | <0,001 | 0.80 | 0.07 | <0,001 |
| **Sex** | 0.01 | 0.11 | 0.942 | -0.66 | 0.13 | <0,001 | -0.08 | 0.08 | 0.338 | -0.07 | 0.09 | 0.426 |
| **Irritable mood*Sex** | 0.09 | 0.09 | 0.314 | -0.19 | 0.10 | 0.052 | -0.16 | 0.08 | 0.034 | -0.14 | 0.10 | 0.154 |
|  |  |  |  |  |  |  |  |  |  |  |  |  |
| **Temper outbursts** | 0.78 | 0.06 | <0,001 | 1.27 | 0.07 | <0,001 | 1.10 | 0.05 | <0,001 | 0.88 | 0.07 | <0,001 |
| **Sex** | 0.20 | 0.09 | 0.035 | -0.54 | 0.12 | <0,001 | -0.07 | 0.06 | 0.287 | -0.01 | 0.08 | 0.887 |
| **Temper outbursts*Sex** | -0.08 | 0.09 | 0.381 | -0.32 | 0.10 | 0.002 | -0.16 | 0.07 | 0.025 | -0.20 | 0.10 | 0.045 |
|  |  |  |  |  |  |  |  |  |  |  |  |  |

**Table S10. Estimated percentage of adolescents with and without irritability, with any type of psychiatric disorder, any internalizing psychiatric disorder, and any externalizing psychiatry disorder by informant and sex.**

|  |  | **Any psychiatric disorder** | | **Any internalizing psychiatric disorder** | | **Any externalizing psychiatric disorder** | |
| --- | --- | --- | --- | --- | --- | --- | --- |
|  |  | **Without irritability** | **With irritability** | **Without irritability** | **With irritability** | **Without irritability** | **With irritability** |
|  |  | ***% (95%CI)*** | ***% (95%CI)*** | ***% (95%CI)*** | ***% (95%CI)*** | ***% (95%CI)*** | ***% (95%CI)*** |
| **Irritable mood by parent report** | | | | | |  |  |
| **Adolescence** | **Males** | 10.5 (8.5, 12.5) | 40 (34.4, 45.5) | 4.1 (2.9, 5.4) | 16.3 (11.9, 20.7) | 3.5 (2.3, 4.7) | 27.6 (22.5, 32.8) |
|  | **Females** | 9.8 (7.9, 11.8) | 39.2 (33.2, 45.3) | 8.2 (6.4, 10) | 30.7 (24.8, 36.6) | 1.4 (0.7, 2) | 16.6 (12.5, 20.6) |
| **Temper outbursts by parent report** | | | | | |  |  |
| **Adolescence** | **Males** | 11.3 (9.3, 13.3) | 53.6 (46.6, 60.6) | 4.7 (3.3, 6) | 21 (15.2, 26.8) | 3.7 (2.6, 4.9) | 41.8 (34.8, 48.8) |
|  | **Females** | 11.7 (9.7, 13.7) | 44.6 (37.6, 51.7) | 10 (8.1, 11.9) | 32.2 (25.3, 39.2) | 1.6 (0.9, 2.3) | 24.1 (18.4, 29.8) |
| **Irritable mood by self-report** | | | | | |  |  |
| **Adolescence** | **Males** | 11.5 (9.2, 13.7) | 24.1 (18.7, 29.5) | 3.6 (2.2, 5) | 12.8 (8.6, 16.9) | 4.8 (3.4, 6.3) | 13.8 (9.6, 18.1) |
|  | **Females** | 10.1 (7.8, 12.5) | 31.2 (26.3, 36.1) | 8.3 (6.2, 10.5) | 27.7 (22.9, 32.5) | 1.5 (0.8, 2.3) | 7.7 (5.2, 10.2) |
| **Temper outbursts by self-report** | | | | | |  |  |
| **Adolescence** | **Males** | 11.9 (9.8, 14) | 29.4 (22.2, 36.7) | 4.1 (2.8, 5.4) | 15.5 (9.7, 21.3) | 4.7 (3.4, 6.1) | 20 (13.7, 26.3) |
|  | **Females** | 12.1 (9.9, 14.3) | 37.3 (30, 44.5) | 10.3 (8.2, 12.4) | 32.1 (25, 39.2) | 1.6 (0.9, 2.3) | 12 (7.8, 16.2) |
|  | | | | | | | |

In childhood, irritability also affected more the prevalence of psychiatric disorders in females than males. For example, irritable mood as reported by parents had a major impact on the prevalence of any psychiatric disorder in females than in males (b=0.37, SE=0.12, *p*=0.002). Likewise, irritable mood (b=0.53, SE=0.15, *p*<0.001) and temper outbursts (b=0.53, SE=0.17, *p*=0.002) had a major impact on the prevalence of any externalizing disorder in females than in males (**Table S11**, **Table S13**).

**Table S11. Estimated percentage of participants with and without irritability, with any type of psychiatric disorder, any internalizing psychiatric disorder, and any externalizing psychiatry disorder by sex in childhood.**

|  |  | **Any psychiatric disorder** | | **Any internalizing psychiatric disorder** | | **Any externalizing psychiatric disorder** | |
| --- | --- | --- | --- | --- | --- | --- | --- |
|  |  | **Without irritability** | **With irritability** | **Without irritability** | **With irritability** | **Without irritability** | **With irritability** |
|  |  | ***% (95%CI)*** | ***% (95%CI)*** | ***% (95%CI)*** | ***% (95%CI)*** | ***% (95%CI)*** | ***% (95%CI)*** |
| **Irritable mood by parent report** | | | | | |  |  |
| **Childhood** | **Males** | 11.1 (9.4, 12.9) | 37.3 (32.7, 42) | 2.5 (1.6, 3.4) | 15.3 (12, 18.6) | 4.1 (3, 5.2) | 24.8 (20.6, 28.9) |
|  | **Females** | 4.5 (3.3, 5.7) | 25.9 (21.2, 30.6) | 1.7 (1, 2.4) | 14.6 (10.8, 18.4) | 0.7 (0.3, 1.1) | 15.6 (11.7, 19.6) |
| **Temper outbursts by parent report** | | | | | |  |  |
| **Childhood** | **Males** | 11.1 (9.4, 12.7) | 43.8 (38.3, 49.3) | 2.7 (1.8, 3.6) | 17.4 (13.6, 21.3) | 4 (2.9, 5) | 30.3 (25.3, 35.2) |
|  | **Females** | 4.8 (3.7, 6) | 30 (24.7, 35.4) | 2.2 (1.4, 3) | 15 (10.8, 19.3) | 0.7 (0.3, 1.1) | 19.4 (14.7, 24.1) |

**Table S12. Results of logistic regressions by informant examining the association between irritable mood/temper outbursts, and their interaction with sex, and psychiatric disorders (any type, any internalizing, and any externalizing) in adolescence.**

|  |  |  |  |  |  |  |  |  |  |
| --- | --- | --- | --- | --- | --- | --- | --- | --- | --- |
|  | **Any disorder** | | | **Any internalizing disorder** | | | **Any externalizing disorder** | | |
| **Parent report** | **B** | **SE** | ***p*-value** | **B** | **SE** | ***p*-value** | **B** | **SE** | ***p*-value** |
| **Irritable mood** | 1.04 | 0.09 | <0.001 | 0.81 | 0.11 | <0.001 | 1.26 | 0.12 | <0.001 |
| **Sex** | 0.07 | 0.25 | 0.785 | 0.74 | 0.27 | 0.007 | -0.87 | 0.36 | 0.016 |
| **Irritable mood*Sex** | -0.08 | 0.14 | 0.568 | 0.04 | 0.14 | 0.798 | 0.03 | 0.16 | 0.831 |
|  |  |  |  |  |  |  |  |  |  |
| **Temper outbursts** | 1.06 | 0.10 | <0.001 | 0.76 | 0.10 | <0.001 | 1.38 | 0.13 | <0.001 |
| **Sex** | 0.18 | 0.22 | 0.401 | 0.85 | 0.25 | <0.001 | -0.79 | 0.33 | 0.016 |
| **Temper outbursts*Sex** | -0.17 | 0.14 | 0.228 | -0.04 | 0.14 | 0.778 | 0.00 | 0.17 | 0.985 |
|  |  |  |  |  |  |  |  |  |  |
| **Self-report** |  |  |  |  |  |  |  |  |  |
| **Irritable mood** | 0.57 | 0.10 | <0.001 | 0.82 | 0.14 | <0.001 | 0.73 | 0.14 | <0.001 |
| **Sex** | -0.48 | 0.27 | 0.076 | 0.67 | 0.36 | 0.058 | -1.63 | 0.45 | <0.001 |
| **Irritable mood*Sex** | 0.35 | 0.15 | 0.020 | 0.15 | 0.18 | 0.392 | 0.36 | 0.21 | 0.085 |
|  |  |  |  |  |  |  |  |  |  |
| **Temper outbursts** | 0.66 | 0.10 | <0.001 | 0.80 | 0.12 | <0.001 | 0.86 | 0.12 | <0.001 |
| **Sex** | -0.08 | 0.20 | 0.709 | 1.04 | 0.27 | <0.001 | -1.32 | 0.35 | <0.001 |
| **Temper outbursts*Sex** | 0.15 | 0.14 | 0.268 | -0.04 | 0.16 | 0.806 | 0.24 | 0.18 | 0.186 |

**Table S13. Results of logistic regressions by informant examining the association between irritable mood/temper outbursts, and their interaction with sex, and psychiatric disorders (any type, any internalizing, and any externalizing) in childhood.**

|  |  |  |  |  |  |  |  |  |  |
| --- | --- | --- | --- | --- | --- | --- | --- | --- | --- |
|  | **Any disorder** | | | **Any internalizing disorder** | | | **Any externalizing disorder** | | |
| **Parent report** | **B** | **SE** | ***p*-value** | **B** | **SE** | ***p*-value** | **B** | **SE** | ***p*-value** |
| **Irritable mood** | 0.86 | 0.07 | <0,001 | 1.06 | 0.11 | <0,001 | 1.09 | 0.09 | <0,001 |
| **Sex** | -1.35 | 0.24 | <0,001 | -0.44 | 0.39 | 0.263 | -2.07 | 0.36 | <0,001 |
| **Irritable mood*Sex** | 0.37 | 0.12 | 0.002 | 0.16 | 0.16 | 0.300 | 0.53 | 0.15 | <0,001 |
|  |  |  |  |  |  |  |  |  |  |
| **Temper outbursts** | 1.00 | 0.07 | <0,001 | 1.00 | 0.10 | <0,001 | 1.20 | 0.09 | <0,001 |
| **Sex** | -0.95 | 0.23 | <0,001 | -0.12 | 0.35 | 0.737 | -1.95 | 0.36 | <0,001 |
| **Temper outbursts*Sex** | 0.16 | 0.13 | 0.205 | 0.02 | 0.16 | 0.922 | 0.53 | 0.17 | 0.002 |
|  |  |  |  |  |  |  |  |  |  |

**Table S14. Results of logistic regressions by informant examining the association between irritable mood/temper outbursts, and their interaction with sex, and external indicators of impairment in adolescence.**

|  | **Parent-reported self-harm** | | | **Self-reported self-harm** | | | **School exclusion** | | | **Use of mental health services** | | |
| --- | --- | --- | --- | --- | --- | --- | --- | --- | --- | --- | --- | --- |
| **Parent report** | **B** | **SE** | ***p*-value** | **B** | **SE** | ***p*-value** | **B** | **SE** | ***p*-value** | **B** | **SE** | ***p*-value** |
| **Irritable mood** | 0.78 | 0.13 | <0.001 | 0.66 | 0.13 | <0.001 | 0.63 | 0.12 | <0.001 | 0.77 | 0.08 | <0.001 |
| **Sex** | 1.23 | 0.35 | <0.001 | 1.43 | 0.34 | <0.001 | -1.00 | 0.42 | 0.018 | 0.23 | 0.18 | 0.199 |
| **Irritable mood*Sex** | -0.21 | 0.17 | 0.198 | -0.30 | 0.17 | 0.066 | -0.11 | 0.22 | 0.622 | -0.07 | 0.11 | 0.524 |
|  |  |  |  |  |  |  |  |  |  |  |  |  |
| **Temper outbursts** | 0.79 | 0.13 | <0.001 | 0.66 | 0.13 | <0.001 | 0.72 | 0.11 | <0.001 | 0.75 | 0.08 | <0.001 |
| **Sex** | 1.20 | 0.31 | <0.001 | 1.33 | 0.29 | <0.001 | -1.23 | 0.39 | 0.002 | 0.16 | 0.16 | 0.313 |
| **Temper outbursts*Sex** | -0.22 | 0.17 | 0.209 | -0.30 | 0.16 | 0.064 | 0.04 | 0.20 | 0.842 | 0.00 | 0.12 | 0.998 |
|  |  |  |  |  |  |  |  |  |  |  |  |  |
| **Self-report** | **B** | **SE** | ***p*-value** | **B** | **SE** | ***p*-value** | **B** | **SE** | ***p*-value** | **B** | **SE** | ***p*-value** |
| **Irritable mood** | 0.98 | 0.17 | <0.001 | 0.67 | 0.16 | <0.001 | 0.38 | 0.16 | 0.016 | 0.57 | 0.10 | <0.001 |
| **Sex** | 0.84 | 0.44 | 0.055 | 0.66 | 0.37 | 0.070 | -1.34 | 0.53 | 0.012 | 0.07 | 0.20 | 0.730 |
| **Irritable mood*Sex** | 0.00 | 0.22 | 0.984 | 0.09 | 0.19 | 0.615 | 0.12 | 0.30 | 0.686 | 0.08 | 0.13 | 0.540 |
|  |  |  |  |  |  |  |  |  |  |  |  |  |
| **Temper outbursts** | 0.61 | 0.17 | <0.001 | 0.57 | 0.12 | <0.001 | 0.65 | 0.14 | <0.001 | 0.56 | 0.09 | <0.001 |
| **Sex** | 0.43 | 0.32 | 0.173 | 0.55 | 0.26 | 0.038 | -1.10 | 0.44 | 0.012 | 0.09 | 0.17 | 0.589 |
| **Temper outbursts*Sex** | 0.33 | 0.20 | 0.110 | 0.26 | 0.15 | 0.092 | -0.02 | 0.25 | 0.931 | 0.14 | 0.13 | 0.291 |

Irritable mood and temper outbursts in childhood were associated with self-harming behaviors, school exclusions, and contact with mental health services, with no apparent sex or informant effects (**Table S15**).

**Table S15. Results of logistic regressions by informant examining the association between irritable mood/temper outbursts, and their interaction with sex, and external indicators of impairment in childhood.**

|  | **Parent-reported self-harm** | | | **Self-reported self-harm** | | | **School exclusion** | | | **Use of mental health services** | | |
| --- | --- | --- | --- | --- | --- | --- | --- | --- | --- | --- | --- | --- |
| **Parent report** | **B** | **SE** | ***p*-value** | **B** | **SE** | ***p*-value** | **B** | **SE** | ***p*-value** | **B** | **SE** | ***p*-value** |
| **Irritable mood** | 0.98 | 0.16 | <0,001 | 1.02 | 0.35 | 0.003 | 1.09 | 0.22 | <0,001 | 0.77 | 0.06 | <0,001 |
| **Sex** | 0.00 | 0.58 | 0.995 | 0.89 | 1.99 | 0.656 | -1.67 | 1.39 | 0.230 | -0.35 | 0.15 | 0.023 |
| **Irritable mood*Sex** | 0.02 | 0.23 | 0.929 | -0.92 | 1.38 | 0.505 | 0.26 | 0.45 | 0.556 | -0.02 | 0.10 | 0.842 |
|  |  |  |  |  |  |  |  |  |  |  |  |  |
| **Temper outbursts** | 0.93 | 0.15 | <0,001 | 1.33 | 0.41 | 0.001 | 0.87 | 0.20 | <0,001 | 0.78 | 0.06 | <0,001 |
| **Sex** | -0.02 | 0.52 | 0.972 | 1.30 | 1.63 | 0.425 | -2.30 | 1.31 | 0.080 | -0.46 | 0.14 | 0.001 |
| **Temper outbursts*Sex** | 0.05 | 0.22 | 0.807 | -1.52 | 1.46 | 0.298 | 0.57 | 0.42 | 0.172 | 0.10 | 0.10 | 0.348 |
